# Molecular Characterization of a Tetraspanin from the Human Liver Fluke, *Opisthorchis viverrini*

**DOI:** 10.1371/journal.pntd.0001939

**Published:** 2012-12-06

**Authors:** Supawadee Piratae, Smarn Tesana, Malcolm K. Jones, Paul J. Brindley, Alex Loukas, Erica Lovas, Veerachai Eursitthichai, Banchob Sripa, Sirikanda Thanasuwan, Thewarach Laha

**Affiliations:** 1 Department of Parasitology, Faculty of Medicine, Khon Kaen University, Khon Kaen, Thailand; 2 School of Veterinary Sciences, University of Queensland, Gatton, Queensland, Australia; 3 Department of Microbiology, Immunology and Tropical Medicine, School of Medicine and Health Sciences, George Washington University, Washington, D.C., United States of America; 4 Centre for Biodiscovery and Molecular Development of Therapeutics, Queensland Tropical Health Alliance, James Cook University, Cairns, Queensland, Australia; 5 Department of Biomedical Sciences, Faculty of Allied Health Sciences, Thammasat University, Pathumthani, Thailand; 6 Tropical Disease Research Laboratory, Department of Pathology, Faculty of Medicine, Khon Kaen University, Khon Kaen, Thailand; 7 Liver Fluke and Cholangiocarcinoma Research Center, Faculty of Medicine, Khon Kaen University, Khon Kaen, Thailand; Yale Child Health Research Center, United States of America

## Abstract

**Background:**

The human liver fluke, *Opisthorchis viverrini*, is designated as a group 1 carcinogen, and is the major risk factor for cholangiocarcinoma in endemic countries throughout Southeast Asia. Proteins in the excretory-secretory products and tegumental surface membranes of the fluke have been proposed to play pivotal roles in parasite survival in the host, and subsequent pathogenesis. These macromolecules are therefore valid targets for the development of vaccines and new drugs to control the infection. Tetraspanins (TSP) are prominent components of the tegument of blood flukes where they are essential for tegument formation, are directly exposed to the immune system, and are major targets for a schistosomiasis vaccine. We propose that similar molecules in the surface membranes of *O. viverrini* are integral to tegument biogenesis and will be efficacious vaccine antigens.

**Methodology/Principal Findings:**

The cDNA sequence encoding *O. viverrini* tetraspanin-1 (*Ov*-TSP-1) was identified and cloned. The *Ov-tsp-1*gene was isolated from a cDNA library. *Ov*-*tsp-1* mRNA was expressed most highly in metacercariae and eggs, and to a lesser extent in juvenile and adult worms. Immunolocalization with adult flukes confirmed that *Ov*-TSP-1 was expressed in the tegument and eggs *in utero*. Western blot analysis of r*Ov*-TSP-1 probed with sera from *O. viverrini*-infected humans and hamsters indicated that both hosts raise antibody responses against the native TSP. Using RNA interference we silenced the expression level of *Ov*-*tsp-1* mRNA in adult flukes by up to 72% by 10 days after delivery of dsRNA. Ultrastructural morphology of adult worms treated with *Ov*-*tsp-1* dsRNA displayed a distinctly vacuolated and thinner tegument compared with controls.

**Conclusions/Significance:**

This is the first report of a tetraspanin from the tegument of a liver fluke. Our data imply that tetraspanins play important structural roles in the development of the tegument in the adult fluke. Potential uses of *O. viverrini* tetraspanins as novel interventions are discussed.

## Author Summary

Liver fluke infection is a fish borne disease that afflicts millions of residents in Thailand and Laos. Infection results from eating undercooked freshwater fish contaminated with larvae of the worm *Opisthorchis viverrini*. Infection can lead to cancer of the bile ducts (cholangiocarcinoma). Indeed, *O. viverrini* is designated as a Group 1 carcinogen by the World Health Organization, i.e. a definitive cause for cancer. Proteins produced at the surface and/or released from this parasite play pivotal roles in maintaining the infection and disease. These proteins are valid targets for development of vaccines and new drugs. Tetraspanins are prominent in the tegument (the surface covering) of parasites closely related to *O. viverrini* where they are exposed to immune responses. Similar molecules on the surface of *O. viverrini* may be vital for the parasite's survival and may make effective vaccines. Here the gene coding for *O. viverrini* tetraspanin-1 (*Ov*-TSP-1) was investigated. We used electron microscopy to show that *Ov*-TSP-1 is expressed in the tegument. We then silenced expression of the gene encoding *Ov*-TSP-1 and showed that this resulted in malformation of the tegument, highlighting the importance of this molecule for parasite development and its potential as a vaccine target.

## Introduction

The human liver fluke *Opisthorchis viverrini* has been classified by the World Health Organization's International Agency for Research on Cancer as a Group 1 carcinogen [1]. Approximately 10 million people in Southeast Asia are infected with this neglected parasite [2], and a further 15–20 million are infected throughout Asia with the closely related *Clonorchis sinensis* [3]. Treatment with praziquantel is effective in eliminating current infections, but rapid re-infection occurs and can be accompanied by severe pathology [4], [5]. The pathogenesis, control and re-emergence of *O. viverrini* infection, particularly in Thailand, and the association of *O. viverrini* infection and bile duct cancer have been reviewed recently [6], [7], [8].

New interventions for long-term prevention, such as a vaccine, are urgently needed. It has been proposed that molecules in the excretory-secretory (ES) products and outer epithelial surfaces of this fluke play key roles in the pathogenesis of opisthorchiasis and mediate the fluke's parasitic existence [9], [10]. We recently characterized the tegument proteome of adult *O. viverrini* and identified those proteins exposed on the surface of live worms using a selective biotinylation approach [11]. Of the transmembrane proteins identified, one shared sequence identity with the tetraspanin family of transmembrane proteins. Tetraspanins contain 4 transmembrane domains and are frequently expressed at the cell surface in association with each other and with other molecules, such as integrins, where they function to regulate cell adhesion, migration, proliferation, and differentiation [12], [13]. Tetraspanins have also been shown to act as receptors for viruses, most notably CD81 binding to hepatitis C [14].

Tetraspanins are prominent on the surface of the intra-mammalian stages of the human blood fluke, *Schistosoma mansoni. Sm*-TSP-2 is a tetraspanin from the tegument of *S. mansoni* [15], [16] that is essential for proper tegument formation, and silencing of the *Sm-tsp-2* gene proved lethal for schistosomula *in vivo* [17]. Indeed, the *S. mansoni* genome contains a large family of tetraspanin-encoding genes that have diverse expression profiles [18], and one of the most highly upregulated genes in developing schistosomula encodes a tetraspanin on the tegument surface [19].

The tegument of *O. viverrini* metacercariae, juvenile and adult flukes is exposed to the mammalian host tissues; indeed the tegument of the adult fluke forms an intimate contact with the host biliary epithelium [20], resulting in chronic cell proliferation, immunopathology and ultimately tumorigenesis [21], [22]. In addition, molecules in the tegument membranes are a major target for the development of new drugs and vaccines against the parasite. The transcriptome [23], [24] and secreted proteome [11] of *O. viverrini* have been characterized, revealing tetraspanins as a major component of the tegument membrane. The tegument in particular plays a crucial role in survival of parasitic flukes and is therefore considered as a target for vaccine development in schistosomiasis [15]. Indeed, *Sm*-TSP-2 from *S. mansoni* is entering Phase I clinical trials [25], and a tetraspanin from the zoonotic *Schistosoma japonicum* (Sj23) is being considered as a vaccine targeting the buffalo reservoir host in an attempt to interrupt transmission to humans [26]. TSPs have recently proven to be efficacious vaccine antigens against cestode parasites [27], highlighting their efficacy in multiple classes of platyhelminths. While less information is available for tegument protein vaccines from liver flukes, efficacy with tegument extracts has been reported for *Fasciola hepatica* [28] and with a recombinant tegument protein from *C. sinensis* [29].

Here, for the first time, we describe the cloning and characterization of a liver fluke tetraspanin, *Ov-tsp-1*, and locate its site of expression to the tegument of adult flukes. Suppression of *Ov-tsp-1* in adult flukes impacts on proper tegument formation and results in increased vacuolation, implying that this protein is essential for fluke development and survival and is therefore worthy of consideration as a vaccine and/or drug target.

## Materials and Methods

### *Opisthorchis viverrini*

Metacercariae of *O. viverrini* were collected from infected fishes obtained from reservoirs in Khon Kaen province, Thailand. Fishes were digested with pepsin as described [23]. Syrian golden hamsters (*Mesocricetus auratas*) were infected with 50–100 metacercariae each via an intragastric tube. Hamsters were maintained at the animal facility, Faculty of Medicine, Khon Kaen University and protocols used for animal experimentation were approved by the Animal Ethics Committee of Khon Kaen University based on the Ethics of Animal Experimentation of the National Research Council of Thailand. Juvenile flukes (2 weeks old) with incompletely developed reproductive organs [30] and adult flukes (6 weeks old) were harvested from gall bladders and bile ducts of hamsters. To collect *O. viverrini* eggs, adult worms were cultured in RPMI supplemented with 1× antibiotics (streptomycin/penicillin, 100 µg/ml) at 37°C under 5% CO_2_ in air. After 18 h incubation, culture medium was collected and centrifuged at 5,000 rpm for 10 min to collect the eggs. Eggs were stored at −70°C until required [31].

### Cloning of *Ov-tsp-1* cDNA

The cDNA encoding the open reading frame (ORF) of *Ov*-*tsp-1* was obtained by PCR from an adult worm cDNA library [23]. Oligonucleotide primers for PCR to amplify the complete ORF were designed based on expressed sequence tags (ESTs) [23], [24]. The primers used were Ov-TSP1F (5′-ATGAGATGATGGGTTGTGTCCAATGC-3′) and Ov-TSP1R (5′-AGTCACTTAAGTTGCTATGGCATAGTCC-3′). PCR reactions were conducted as follows: 100 ng of *O. viverrini* cDNA library as template, 0.2 mM dNTP, 1.5 mM MgCl_2_ performed with 1 unit *Taq* polymerase (Invitrogen, Germany) with 35 cycles of denaturation at 95°C for 1 min, annealing at 60°C for 1 min, extension at 72°C for 2 min and a final extension at 72°C for 10 min. PCR products were identified by agarose gel electrophoresis and purified by gel extraction with a commercial gel extraction kit (Fermentas, EU). PCR products were sequenced before ligation into the pGEM T Easy vector (Promega, USA), and this construct was used to transform *E. coli* JM109 competent cells (Promega). Sequences were subjected to BLAST searching against the GenBank database. Recombinant clones were screened for ampicillin resistance and blue/white selection, and sequence inserts confirmed by PCR amplification using oligonucleotide primers corresponding to the multiple cloning site promoter sequences, T7 and SP6. White colonies were selected, and insert sequences determined using the BigDye terminator method (1^st^ BASE, Singapore).

### Sequence analysis

DNA sequences were evaluated using BioEdit V7.0.5 [32]. The edited sequences were translated to protein using web based translation software at http://bio.lundberg.gu.se/edu/translat.html and compared to related sequences using the basic local alignment search tool (BLAST) at http://blast.ncbi.nlm.nih.gov/ [33]. Signal peptide analysis was conducted with the SignalP 3.0 Server at http://www.cbs.dtu.dk/services/SignalP/. Multiple sequence alignments were compiled using ClustalW in the BioEdit program. Transmembrane regions were predicted using the TMpred server at http://www.ch.embnet.org/software/TMPRED_form.html.

### Phylogenetic analysis

Phylogenetic relationships among *Ov*-TSP-1 and TSPs from a range of organisms was constructed based on amino acid sequences. ORFs were aligned using ClustalW [34]. A phylogenetic tree was constructed with p-distance matrix using the neighbor-joining method [35] with 1,000 bootstrap samplings in the MEGA software package version 4.0.2 [36], [37].

### Cloning and recombinant protein production of the large extracellular loop region of *Ov*-TSP-1

The nucleotide sequence corresponding to the large extracellular loop (LEL) region of *Ov*-TSP-1 (amino acid residues 106 to 202) was identified using TMpred and amplified by PCR with the forward primers TSP1_EC2F_pET, 5′-AGC**CATATG**GGCTATGTGTTCCGGGAG and the reverse primer TSP1_EC2R_pET, 5′-AGC**GGATCC**CTACTTGTCCTTGAAGAATCG that incorporated an *Nde* I site at the 5′ end and a *Bam* HI site at the 3′ end (underlined) to ensure in-frame fusion with the vector-derived 6× His epitope at the N-terminus of the pET-15b expression vector (Novagen, USA). PCR products were sub-cloned into pGEM-T. Recombinant plasmids were then digested with *Nde* I and *Bam* HI, and *Nde* I/*Bam* HI fragments cloned into pET-15b to produce plasmid pLEL-*Ov*-TSP-1; the identity and in-frame fusion to the 6× His tag of the insert was confirmed by sequencing.

BL21DE3 strain *E. coli* (Novagen) was transformed with pLEL-*Ov*-TSP-1. Transformed bacteria were induced with 1 mM IPTG in LB medium for 3 hr at 37°C on a shaking platform to produce recombinant LEL-*Ov*-TSP-1. Recombinant LEL-*Ov*-TSP-1 was purified by affinity chromatography (His•Bind Resin, Novagen) under denaturing condition with 6M urea. The protein was refolded by dialysis against PBS and analyzed by Coomassie stained SDS-PAGE.

### Synthesis of double-stranded RNAs (dsRNAs)

dsRNAs derived from either *Ov-tsp-1* or firefly luciferase (LUC) were prepared from plasmid DNA using a MEGAscript RNAi Kit (Ambion), following the manufacturer's instructions. dsRNA targeting *Ov-tsp-1* was amplified from a plasmid (above) using primers flanked with T7 RNA polymerase promoter sequence (underlined) at the 5′ ends. The *Ov*-*tsp-1* dsRNA of 519 bp (residues 133–651 of the transcript, GenBank accession JQ678706) was generated using primers ds-TSP1_T7-F, 5′ TAATACGACTCACTATAGGGGCGTCCGGACACTATG and ds-TSP1_T7-R, 5′ TAATACGACTCACTATAGGGCTCGAAGGCGGCAATTGAC. The PCR conditions were 35 cycles of denaturation at 95°C for 30 sec, annealing at 60°C for 30 sec, extension at 72°C for 1 min, final extension at 72°C for 10 min. An irrelevant negative control, *luciferase* dsRNA derived from pGL3-basic (www.promega.com), was amplified using primers ds-LUC_T7-F5′ TAATACGACTCACTATAGGG TGCGCCCGCGAACGACATTTA and ds-LUC_T7-R5′ TAATACGACTCACTATAGGG GCAACCGCTTCCCCGACTTCCTTA [38]. Integrities of the dsRNAs were assessed on a 1% agarose gel and concentrations determined by spectrophotometer (NanoVue, GE Healthcare, USA).

### Delivery of dsRNA by electroporation

Adult worms were washed prior to electroporation [31]. Thirty worms in each group (4 groups) were resuspended in 100 µl of culture medium (1× RPMI-1640, 1× antibiotic/antimycotic, 1% glucose, 1 mM E 64) supplemented with 50 µg *Ov-tsp-1* or *luc* dsRNAs in 4 mm gap cuvettes (Bio-Rad, Hercules, CA, USA) and exposed to single square wave electroporation at 125 V with 20 ms duration (Electroporator Gene Pulser Xcell, Bio-Rad). After pulsing, worms were maintained in culture medium supplemented with 2 µg dsRNA at 37°C under 5% CO_2_ in air. Worms were soaked in 2 µg dsRNA for 16 days with changes of media containing dsRNA every second day. Parasites were harvested at days 1, 3, 6, 10 and 16. Moreover, dsRNA-treated adults parasite were sampled on days 1, 3 and 6 and fixed in 3% glutaraldehyde in 0.1 M phosphate buffer at pH 7.4 for transmission electron microscopy.

### RNA extraction and real time quantitative RT-PCR

Total RNA of adult and juvenile flukes, metacercariae and eggs of *O. viverrini* was extracted in TRIZOL (Invitrogen, USA). Concentrations of RNA were determined with a spectrophotometer. Real time qRT-PCR (qPCR) was performed to detect expression of *Ov-tsp-1 O. viverrini*. First strand cDNA was synthesized from 1 µg of DNase treated RNA using a cDNA synthesis kit (Fermentas). The *Ov-tsp-1* specific primers spanned nt 1- 192. The primers were TSP1_EXF 5′-ATGATGGGTTGTGTCCAATGC-3′ and TSP1_EXR 5′-ACCGCCGACTCCCATGAGAGC-3′. The SYBR Green reagent was used for qPCR with a Mx3005P Real-time-PCR System (STRATAGENE, USA). SYBR Green reactions consisted of 6.25 µl of 2×Brilliant SYBR Green QPCR Master Mix (STRATAGENE), 0.75 µl (10 mM) of forward primer and reverse primer, 0.1875 µl of reference Dye (ROX; 1∶200), 100 ng of first-stand cDNA and sterile water to a final volume of 12.5 µl. Duplicate reactions were carried out, as follows: initiation pre-heat for one cycle at 95°C for 10 min followed by 40 cycles of denaturation at 95°C for 30 sec, annealing at 55°C for 30 sec and extension at 72°C for 1 min. The expression of candidate mRNAs was measured using actin mRNA as a constitutively expressed control. To evaluate transcript levels in adult worms exposed to dsRNAs, total RNA was extracted from individual worms using TRIZOL reagent and contaminating genomic DNA was removed by DNase I. qPCR was performed using an ABI7500 thermal cycler using the SYBR Green assay; triplicate samples were included in each group. PCR reactions consisted of 12.5 µl of SYBR Green Master Mix (TAKARA Perfect Real-time Kit), 0.5 µl (10 mM) of forward primer and reverse primers, 0.5 µl of reference Dye (ROX), 1 µl (equivalent to 50 ng of total RNA) of first- stand cDNA and water to a final volume of 25 µl. PCR cycling conditions were initiation pre-heat for one cycle at 95°C for 10 minutes followed by 40 cycles of denaturation at 95°C for 30 seconds, annealing at 55°C for 30 seconds and extension at 72°C for 45 seconds. Expression levels of the *Ov-tsp-1* and actin mRNAs (OvAE1657, GenBank EL620339.1) [23] were determined. The mRNA expression level of *Ov-tsp-1* (or LUC) was normalized with actin mRNA and presented as the unit value of 2^−ΔΔCt^ where ΔΔCt = ΔCt (treated worms) −ΔCt (non-treated worms) [39]. Data are presented as the mean ± standard error. Differences between groups were assessed using Student's *t*-test (GraphPad Prism Software, www.graphpad.com); *p*≤0.05.was considered statistically significant.

### Production of antiserum

Anti-r*Ov*-TSP-1 serum was produced in BALB/c mice. For the first immunization, purified recombinant *Ov*-TSP-1 (100 µg per mouse per immunization) was emulsified in Freund's complete adjuvant (Sigma-Aldrich, St. Louis, MO, USA) and subcutaneously injected. Mice were boosted twice at two weekly intervals using the same quantity of protein formulated with Freund's incomplete adjuvant (Sigma-Aldrich). Blood was collected from each mouse before immunization and again at two weeks after the final immunization.

### Western blot analysis

An immunoblot assay was performed to identify anti-*Ov*-TSP-1 serum antibodies in naturally infected humans and experimentally infected golden Syrian hamsters determined to be positive for *O. viverrini* infection by fecal microscopy. Samples were from a pre-existing collection of de-identified sera (the protocol had been approved by The Khon Kaen University Ethics Committee for Human Research based on the declaration of Helsinki and the ICH Good Clinical Practice Guideline). Recombinant *Ov*-TSP-1 was resuspended in denaturing buffer, boiled for 5 min, and run on a 17% SDS-PAGE gel. Proteins were transblotted onto nitrocellulose membrane (Mini Trans-Blot Cell, Bio-Rad). The membrane was cut into strips, each strip containing 6 µg of recombinant *Ov*-TSP-1. The strips were washed with PBST (1× PBS + 0.01% Tween-20) for 5 min then blocked for 1 h with blocking buffer (5% skimmed milk in PBST). Strips were incubated with sera from patients and hamsters. Non-infected hamsters served as negative controls. Sera from mice immunized with recombinant *Ov*-TSP-1 were used as positive controls. All sera were diluted at 1∶50 in antibody buffer (2% skimmed milk in PBST) and incubated overnight at 4°C with shaking. Strips were washed twice with PBST for 10 min followed by incubation for 2 h with HRP-goat anti-human IgG, HRP-goat anti-hamster IgG and HRP-goat anti-mouse IgG (diluted 1∶1,000 in antibody buffer). The strips were washed again with PBST twice for 10 min and color reactions were detected by adding 3, 3′-diaminobenzidine (DAB) substrate.

### Immunohistochemistry

Sections containing adult *O. viverrini* were de-paraffinized using xylene then rehydrated in an ethanol series, 100%, 90%, 80% and 70% ethanol, 5 min each. Sections were immersed in citrate buffer (pH 6) and autoclaved for 10 min for antigen unmasking, followed by blocking with 3% H_2_O_2_ in methanol. Thereafter they were incubated overnight at 4°C in mouse anti-*Ov*-TSP-1 sera diluted 1∶200 in PBS. Sections were probed with goat-anti-mouse IgG-HRP (Invitrogen, USA) diluted 1∶1,000 dilution in PBS. Peroxidase reaction products were visualized with 3,3′-diaminobenzidine (DAB) (Sigma-Aldrich). Counterstaining was performed with Mayer's hematoxylin for 5 min. A positive signal was indicated by a reddish-brown color under light microscopy.

### Transmission electron microscopy (TEM)

Adult worms, electroporated with 2 µg/ml of *Ov-tsp-1* or *luciferase* dsRNAs and cultured for 7 days at 37°C under 5% CO_2_ atmosphere, were washed then fixed in 3% glutaraldehyde in 0.1 M phosphate, pH 7.4, followed by fixation in potassium ferricyanide-reduced osmium tetroxide. Fixed worms were dehydrated in acetone and embedded in Epon Resin (ProSciTech, Australia). Ultrathin sections were mounted onto copper grids, contrasted in uranyl acetate and lead citrate and examined using a JEM 1011 transmission electron microscope (Jeol) operated at 80 kV and equipped with a digital camera.

### GenBank accession number

The sequence of the transcript coding for *O. viverrini* tetraspanin-1 has been assigned GenBank accession number JQ678706.

## Results

### General characteristics of *Ov-tsp-1*

**Figure 1:**
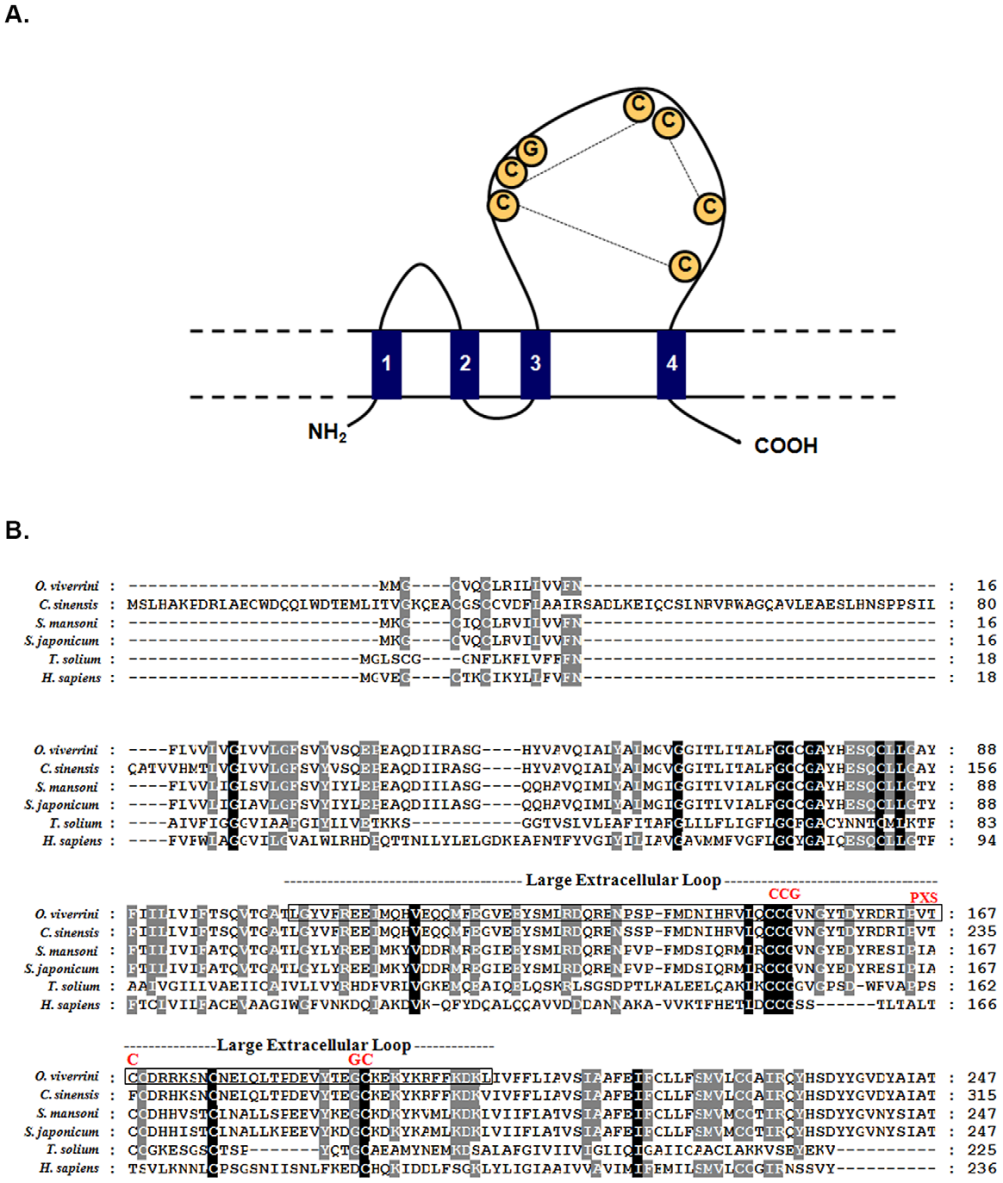
Sequence and structure of *Ov*-TSP-1, a tetraspanin from *Opisthorchis viverrini*. Panel A: Schematic illustration of the structural design of *Ov-*TSP-1. Panel B: Multiple sequence alignment of the deduced amino acid sequence of *Ov*-TSP-1 with other members of the tetraspanin superfamily. Large extracellular loops are enclosed in boxes. Identical residues of sequences are shown in black boxes, conserved substitutions in gray boxes.

**Figure 2:**
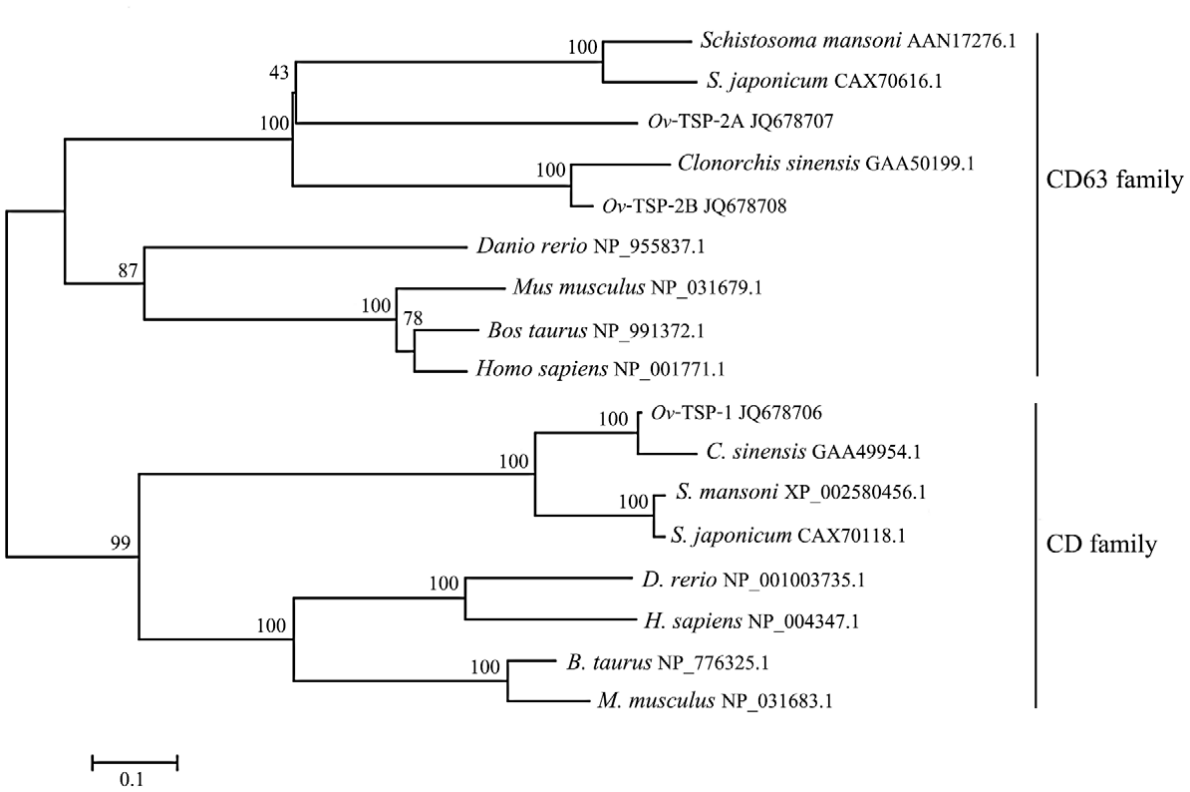
Phylogenetic analysis of tetraspanins from *Opisthorchis viverrini* and homologs from related superfamilies. *O. viverrini* tetraspanins can be grouped within extant two families, the CD family and CD63 family. CD family: *Clonorchis sinensis* (GAA49954.1), *Schistosoma mansoni* (XP_002580456.1), *S. japonicum* (CAX70118.1), *Danio rerio* (NP_001003735.1), *Bos taurus* (NP_776325.1), *Mus musculus* (NP_031683.1), *Homo sapiens* (NP_004347.1). CD63 family: *C. sinensis* (GAA50199.1), *S. mansoni* (AAN17276.1), *S. japonicum* (CAX70616.1), *D. rerio* (NP_955837.1), *B. taurus* (NP_991372.1), *M. musculus* (NP_031679.1), *H. sapiens* (NP_001771.1). Note that the mouse, bovine and human proteins presented on the CD clade are distinct proteins from those shown in the CD63 clade.

A full-length cDNA sequence encoding the first CD9-like tetraspanin from a liver fluke is described, and was designated, *Ov-tsp-1*. The sequence was assigned GenBank accession number JQ678706. The open reading frame consists of 744 base pairs encoding putative protein of 247 amino acids. *Ov*-TSP-1 contains four transmembrane domains, a short extracellular loop (EC1 – 24 amino acids), a very short intracellular loop (4 amino acids), and a large extracellular loop (LEL) or extracellular loop 2 (EC2 – 97 amino acids), flanked by a relatively short N-terminal and C-terminal cytoplasmic tails (9, 25 amino acids) (Figure 1A). The LEL is subdivided into a constant region (containing α helices A, B and E), and a variable region, containing various protein–protein interaction sites (Figure 1A and 1B). A secretory signal peptide was predicted from the deduced amino acid sequence with a putative cleavage site located between amino acids 30 and 31 (GFS-VY). *Ov*-TSP-1 showed the conserved characteristics of the TSP family, notably the signature cysteine–cysteine-glycine (CCG) motif in the LEL, which is the location for the formation of three disulfide bonds within the LEL and influences interactions with other molecules (Figure 1). The deduced amino acid sequence comparison between *Ov*-TSP-1 and other tetraspanins from various organisms in GenBank protein databases showed that *Ov*-TSP-1 shares 97% identity with a TSP of its close relative, *Clonorchis sinensis* (GAA49954.1) calculated from the alignment over 229 amino acid (amino acid position 19–247 of *Ov*-TSP-1 to 87–315 of *C. sinensis*), and 74% identity with *Sm*-TSP-1 of *Schistosoma mansoni* (XP_002580456.1) (Figure 1). Phylogenetic analysis of *Ov*-TSP-1 and related TSPs was drawn based on amino acid sequences presences in 2 clades; CD and CD63 families. *Ov*-TSP-1 is in the largest cluster of the tetraspanin family, the CD family [40]. It is grouped together with *C. sinensis* (GAA49954.1) while *S. mansoni* and *S. japonicum* CD9-like proteins are classified in a sister group (Figure 2).

### *Ov-tsp-1* is expressed throughout the developmental cycle of *O. viverrini*

**Figure 3:**
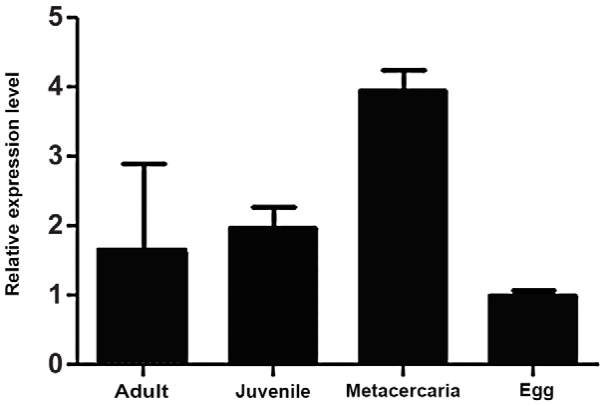
Expression of *Ov-tsp-1* in different developmental stages of *O. viverrini*. RNA levels relative to the gene encoding actin, *Ov-actin*, were analyzed by real-time qRT-PCR. Transcript levels were calculated from duplicate specimens from each treatment group and the data are presented as means ± SD.

The expression profile of *Ov-tsp-1* in the different developmental stages of *O. viverrini* was examined by qPCR using RNAs isolated from adult flukes, two-week old juvenile flukes, metacercariae and eggs. *Ov-tsp-1* is expressed in all stages; expression was highest in metacercariae followed by two-week old juveniles, adults and eggs in descending order (Figure 3). Expression levels of the actin gene of *O. viverrini*, *Ov-actin*, employed here as an internal control, appeared to be similar and unchanged among developmental stages.

### *Ov*-TSP-1 is expressed in the tegument of adult *O. viverrini*

**Figure 4:**
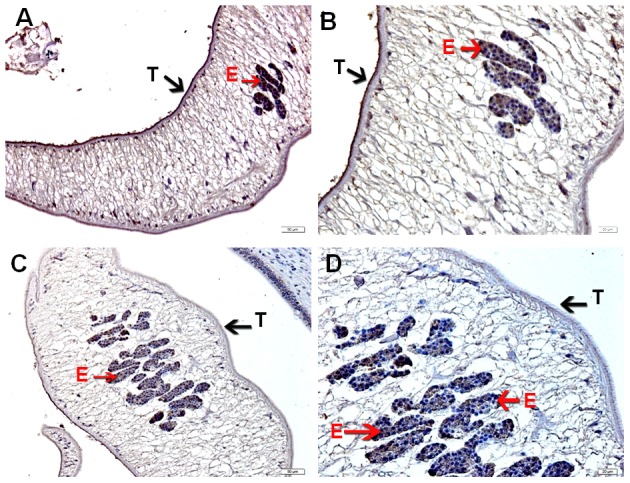
Immunohistochemical detection of *Ov-*TSP-1 in tissue sections of adult *O. viverrini* in the bile ducts of an infected hamster. (A–B) Mouse anti-*Ov*-TSP-1 IgG bound to the tegument (T) and parasite eggs (E). (C–D) Control serum from the same mouse prior to immunization did not bind to the same structures. Scale bars are shown.

**Figure 5:**
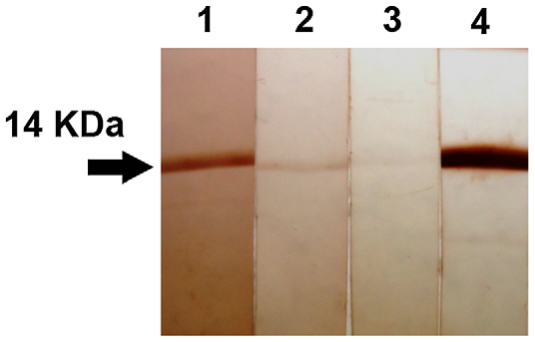
Western blot analysis for recognition of *Ov-*TSP-1 by sera from *O. viverrini* infected humans and hamsters. Lanes 1, immunoblot of r*Ov*-TSP-1 probed with serum of *O. viverrini* infected human; lane 2, immunoblot of r*Ov*-TSP-1 probed with serum of *O. viverrini* infected hamster; lane 3, immunoblot of r*Ov*-TSP-1 probed with normal serum of hamster and lane 4, immunoblot of r*Ov*-TSP-1 probed with sera of mice immunized with r*Ov*-TSP-1.

The recombinant LEL domain of *Ov*-TSP-1 (residues 106–202; r*Ov*-TSP-1) produced in *E. coli* was predominantly found in the insoluble pellet, and required 6 M urea to solubilize. The recombinant protein was purified using nickel chelate affinity chromatography under denaturing conditions, and desalted using Ultra-15 Centrifugal Filter cartridges. SDS-PAGE and immunoblot analysis of the recombinant protein showed migration of the expected molecular mass (∼14 kDa). Purified and desalted r*Ov*-TSP-1 was used to immunize mice. Anti-*Ov*-TSP-1 serum localized *Ov*-TSP-1 to the tegument of adult worms in the liver of hamsters infected with *O. viverrini*. Pre-immunization serum did not stain any *O. viverrini* tissues (Figure 4). Immunoblot analysis showed that sera from humans and hamsters infected with *O. viverrini* both reacted with r*Ov*-TSP-1 (Figure 5).

### dsRNA mediated knockdown of *Ov-tsp-1* expression

**Figure 6:**
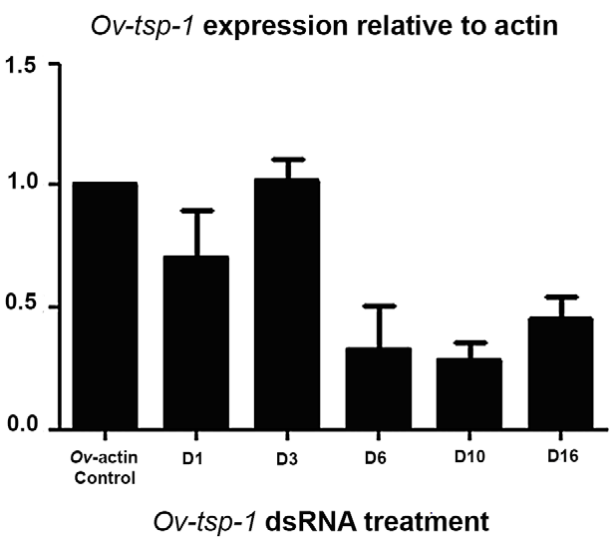
Suppression of *Ov-tsp-1* mRNA in adult *O. viverrini* by RNA interference (RNAi). Real time qRT-PCR analysis of *Ov-tsp-1* transcription levels relative to *Ov-actin* (mean± standard error) showing reduction in expression of *Ov-tsp-1* mRNA in adult *O. viverrini* on days (D) 1–16 of ds-*Ov-tsp-1* treatment by electroporation and soaking. Transcript levels were calculated in triplicate from three randomly-selected parasites from each treatment group and the data are presented as means ± SD. Student's *t*-tests confirmed significant differences as indicated.

dsRNA of *Ov-tsp-1* was introduced into adult worms by square wave electroporation followed by soaking to mediate knockdown of *Ov-tsp-1* via the RNAi pathway. This RNAi procedure knocked down expression of *Ov-tsp*-1 by 67% (*p* = 0.06), 72% (*p* = 0.01) and 55% (*p* = 0.02) on days 6, 10 and 16 *in vitro*, respectively, compared to the negative control group that received *luciferase* dsRNA (Figure 6). The cultured flukes were visually monitored for viability on a daily basis; no differences were evident among treatment groups (not shown).

### Suppression of *Ov*-*tsp-1* mRNA results in malformation of the tegument

**Figure 7:**
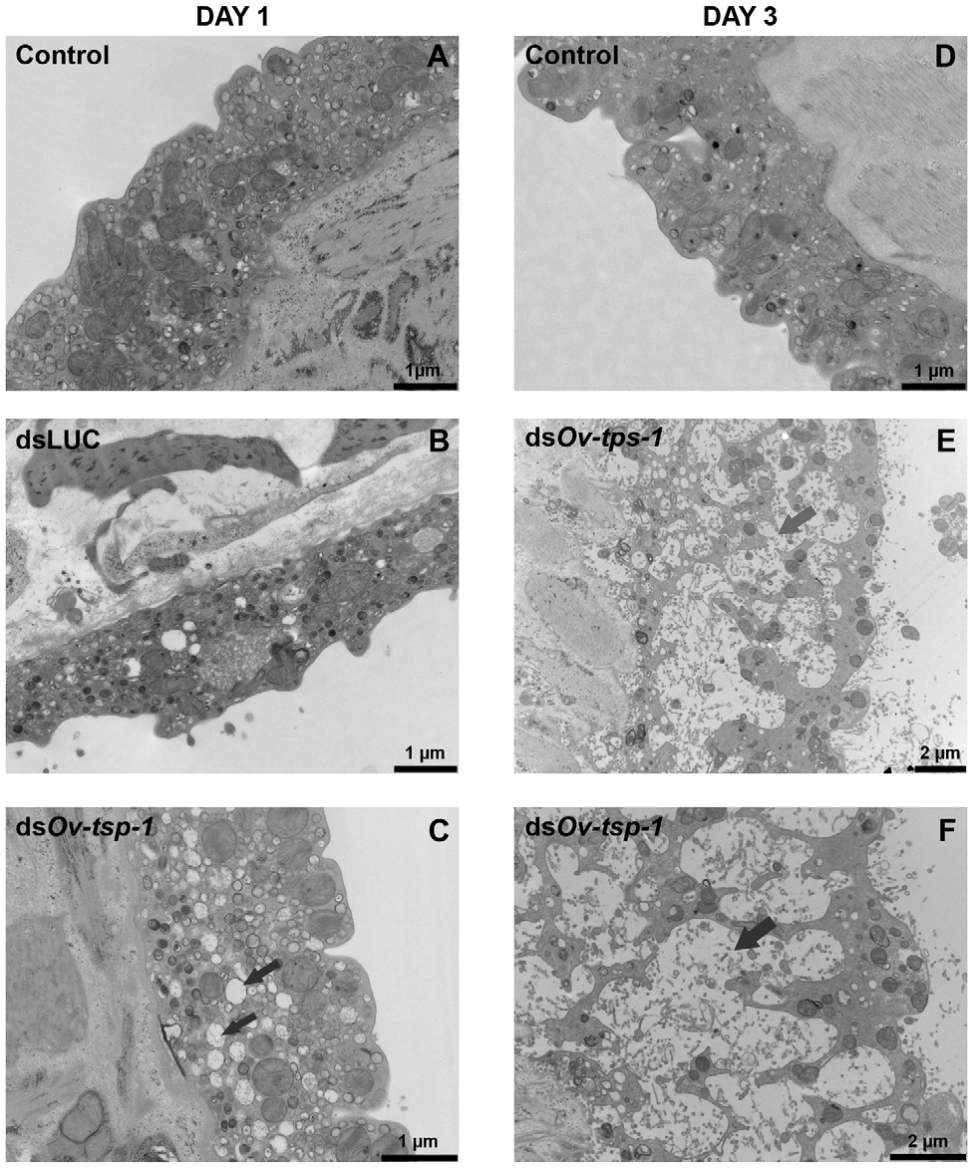
Ultrastructure of the tegument of adult *O. viverrini* treated with *Ov-tsp-1* dsRNA RNA observed using transmission electron microscopy. Panel A = Tegument of non-treated adult *O. viverrini* in RPMI medium for 1 day. Panel B = Tegument of adult *O. viverrini* treated with firefly *luciferase* dsRNA for 1days. Panel C = Tegument of adult *O. viverrini* treated with *Ov-tsp-1* dsRNA for 1days. Panel D = Tegument of non-treated adult *O. viverrini* in RPMI medium for 3 days. Panel E and F = Tegument of adult *O. viverrini* treated with *Ov-tsp-1* dsRNA for 3 days. The tegument of knocked down *tsp-1* worm is more highly vacuolated (indicated by arrows) and thinner compared with controls.

To investigate the effect of silencing *Ov*-*tsp-1* expression on the formation of the *O. viverrini* tegument, adult flukes that had been exposed to *Ov-tsp-1* dsRNA *in vitro* were visualized by TEM. *Ov-tsp-1* dsRNA-treated worms cultured for one to three days displayed an anatomically dissimilar tegument structure to that from control worms exposed to *luciferase* dsRNA (Figure 7). The tegument of *Ov-tsp-1* dsRNA treated worms (Figure 7C, E and F) was more highly vacuolated than firefly *luciferease* dsRNA and non dsRNA controls (Figure 7B, A and D), with extensive and enlarged vacuoles throughout the surface layer.

## Discussion

Tetraspanins (TSPs) are a family of membrane-spanning proteins that display four hydrophobic transmembrane domains interspersed with two extracellular loops and short intracellular amino and carboxyl tails [41], [42], [43]. TSPs are found in all multicellular eukaryotes where they orchestrate the tetraspanin web, an association of different transmembrane proteins (including other TSPs) to stabilize cell membranes and coordinate intracellular and intercellular processes such as signal transduction, cell proliferation, adhesion, migration, fusion and even host-pathogen interactions [44], [45], [46]. Although they have broad functional importance, defined roles for most mammalian TSPs remain elusive. Many recent studies have therefore focused on structure and/or functional relationships of TSPs from vertebrates. Herein we describe *Ov*-TSP-1, the first tetraspanin from the carcinogenic liver fluke, *O. viverrini*, and provide the first functional analysis utilizing gene silencing approaches for a TSP from any liver fluke.

*Ov*-TSP-1 showed the typical TSP structure including the signature CCG motif, which is the main point for the formation of two to four disulfide bridges with additional cysteine residues at fixed positions within the LEL. Members of the TSP family normally have four to six conserved extracellular cysteines forming two to three disulfide bonds [45], [47], [48]. *Ov*-TSP-1 consists of six cysteines linked into three putative disulfide bonds. The four cysteine motifs are greatly derived in the metazoan tetraspanins and more importantly, that the reduction of cysteines in tetraspanins has been a recurring trend in the evolution of tetraspanins [49]. Several TSPs from trematodes have been discovered in the tegumental membranes, including *S. mansoni* TSP-1 and TSP-2 [15], Sm07392 [16], [50] and Sm23 [51]. More recently a TSP was identified using proteomics from the surface of the liver fluke *Fasciola hepatica* [52].

Immunolocalization revealed that *Ov*-TSP-1 is distributed throughout the membranes of the tegument of adult worms and eggs in the uterus. *Ov*-TSP-1 is recognized by sera from *O. viverrini*-infected humans and hamsters, indicating that the LEL is accessible to antibodies and is indeed immunogenic during natural infection. Moreover, *Ov-tsp-1* mRNA is expressed throughout the life cycle of *O. viverrini*, implying that the TSP-1 protein is expressed by the different intra-mammalian developmental stages and would therefore be continuously presented to the immune system from excystation of metacercariae to maturation of adult worms. *S. mansoni* expresses a family of more than 20 TSPs that display diverse expression profiles throughout the schistosome's development [18]. Indeed one of these, *Sm-tsp-3*, is accessible on the surface of live adult *S. mansoni* [16] and is the most highly upregulated mRNA in maturing schistosomula [19].

Many TSPs execute their functions through interactions with integrins. These interactions are important for integrin-mediated cell adhesion to the extracellular matrix. In addition, tetraspanins can play roles in intracellular transport, signal transduction, cell proliferation, adhesion, migration and fusion [46]. As such, they have been implicated in diverse pathologic processes such as inflammation, lymphocyte activation and cancer [43]. It is noteworthy that some mammalian immune cell surface TSPs act as receptors for pathogens. CD81 is required for internalization of bacteria such as *Listeria monocytogenes* [53]. CD81 also acts as a receptor for hepatitis C virus, and neutralizing anti-HCV antibodies inhibit virus binding to the LEL of CD81 [54].

TSPs are well represented in invertebrate genomes but to date little is known about their function. The free-living nematode, *Caenorhabditis elegans*, expresses a TSP in its outer surface, the cuticle, where it plays a critical role in maintenance of epithelial cell integrity. Silencing of the gene is lethal during molting [55]. To date, only one study has addressed the function of a TSP from a parasitic helminth from any phylum; Tran and co-workers demonstrated that silencing of the *Sm-tsp-2* gene by RNAi in both larval and adult intra-mammalian stages of *S. mansoni* resulted in a significantly thinner and distinctly vacuolated tegument and morphology consistent with a failure of tegumentary invaginations to close [17].

To determine whether *O. viverrini* TSPs might perform essential roles in the formation and stability of the tegument of liver flukes, we used RNAi to silence the expression of *Ov-tsp-1* in the adult stage of the parasite. RNAi has been successfully utilized to silence gene expression in liver flukes. McGonigle *et al*. [56] used RNAi to silence cathepsin B and L gene expression in newly excysted juveniles (NEJs) of *F. hepatica* and showed a corresponding reduction in target transcript levels and reduction in the encoded proteins in the gut. RNAi of either enzyme in NEJs induced transient, abnormal locomotion phenotypes, and significantly reduced penetration of the rat intestinal wall. We recently showed that Cy3-labeled small RNAs could be introduced into adult *O. viverrini* by square wave electroporation, and subsequently identified the RNAs in the parenchyma, gut and reproductive organs [31]. We electroporated dsRNA targeting the protease cathepsin B into adult flukes which resulted in a significant reduction in specific mRNA levels and cathepsin B enzymatic activity [31]. Here we show that expression of *Ov-tsp-1* mRNA was suppressed in adult flukes by square wave electroporation-mediated RNAi and we identified a tegument malformation phenotype for the first time for any liver fluke. RNAi targeting *Ov*-*tsp-1* resulted in deformities of the tegument of adult worms within 24 hours of exposure to the dsRNA. These findings indicated a role for *Ov*-TSP-1 in biogenesis of the tegumental cell membrane and maintenance of structural integrity and, when combined with its recognition by antibodies from infected mammalian hosts, justify further exploration of this antigen as a target for the development of new therapeutics against opisthorchiasis.

Tetraspanins from other platyhelminths, including schistosomes and tapeworms, display protective efficacy when deployed in recombinant form as experimental vaccines [15], [26], [27], [57], [58], [59]. More specifically, not only is the schistosome tetraspanin *Sm*-TSP-2 selectively recognized by IgG1 and IgG3 antibodies of persons naturally resistant to *S. mansoni* infection, recombinant TSPs of *S. mansoni* elicit significant protection against challenge infection in mice [15]. Second, investigation of the extracellular loop of the tetraspanin T24 of *Taenia solium* revealed that it provided, in Western blot analysis, marked sensitivity (94%) and specificity (98%) in detecting cases of human cysticercosis with two or more viable cysts [59]. Based on these findings with these platyhelminth parasite orthologues and the present findings, *Ov*-TSP-1 can now be considered as a target at which to develop and target a subunit vaccine against human opisthorchiasis and associated cholangiocarcinoma. In addition, given that *Ov*-TSP-1 is recognized by sera of infected humans and hamsters, its utility as a serodiagnostic warrants further investigation. Finally, localization at the surface of a fluke that resides in the mammalian biliary tree, bathed in bile, indicates that analysis of interactions of *Ov*-TSP-1 with its adjacent receptors, signaling proteins and/or structural components of the parasite's tegument will lead to deeper understanding not only of the anatomic and developmental biology of this carcinogenic fluke but also of adaptions to parasitism in an ostensibly inimical niche.
